# Comparative efficacy and acceptability of selective serotonin reuptake inhibitor antidepressants for binge eating disorder: A network meta-analysis

**DOI:** 10.3389/fphar.2022.949823

**Published:** 2022-09-06

**Authors:** Hanying Duan, Lijun Zhu, Min Li, Xinyue Zhang, Beilin Zhang, Shaokuan Fang

**Affiliations:** ^1^ Department of Neurology, Neuroscience Centre, The First Hospital of Jilin University, Changchun, China; ^2^ China-Japan Union Hospital of Jilin University, Changchun, China

**Keywords:** selective serotonin reuptake inhibitor, antidepressants, binge eating disorder, efficacy, acceptability, network meta-analysis

## Abstract

**Background:** There are several selective serotonin reuptake inhibitor (SSRI) antidepressants currently used to treat binge eating disorder (BED), but the efficacy and acceptability of these antidepressants are still controversial. Therefore, we designed a network meta-analysis (NMA) to compare the efficacy and acceptability of different SSRI antidepressants for the treatment of BED.

**Methods:** Four databases including PubMed, Embase, the Cochrane Library, and Web of Science were systematically searched for the eligible randomized controlled trials (RCTs) for the treatment of patients with BED. The analysis was performed with Stata16 software.

**Results:** 9 RCTs were included in this NMA. The results of the study showed that compared with placebo, sertraline and fluoxetine could significantly reduce the frequency of binge eating. Fluoxetine was shown to be the drug with the greatest reduction in Hamilton Rating Scale for Depression (HAMD) score. Besides, all SSRI antidepressants were ineffective in losing weight. In addition, all the investigated antidepressants were found to be well acceptable in regards to the acceptability reflected by the dropout rate.

**Conclusion:** As far as both efficacy and acceptability were concerned, fluoxetine might be the best choice.

## Introduction

It was the first time that Binge eating disorder (BED) was recognized as a diagnosable condition in the Diagnostic and Statistical Manual of Mental Disorders Fifth Edition (DSM-V) (Sarmiento and Lau, 2020). BED is described by recurrent (at least once a week for 3 months), brief (no more than 2 hours) binge eating episodes with feelings of guilt, self-blame and anxiety. In addition to the binge-eating episodes, the DSM-V also requires that an individual must endorse at least three out of five behavioral characteristics: eating food much faster than normal, eating food more than normal, eating food even when they are uncomfortably full, hiding food or eating alone to avoid embarrassment, or being ashamed, depressed, or/and guilty after overeating. Besides, patients with BED are not associated with unhealthy strategies to prevent weight gain, such as fasting, fad dieting and excessive exercise, unlike bulimia nervosa (BN) (Bohon, 2019).

BED is the most common eating disorder and about 1–3 percent of the general population is affected (Hudson et al., 2007; Kessler et al., 2013). It is more common in women (3.5%) and obese individuals (5–30%) (Spitzer et al., 1993; Bruce and Wilfley, 1996). Compared to women without BED (Lloyd-Richardson et al., 2000), women patients with BED usually have a higher body weight and more frequent weight oscillations (Hudson et al., 2007). Besides, BED patients are more likely to be seriously overweight (Hudson et al., 2007; Kessler et al., 2013) and more common to suffer from mental health problems. Almost 80% of BED patients have suffered from another mental health disorder, such as depression, anxiety disorder, alcohol or substance use disorder, obsessive-compulsive disorder (OCD) (Roehrig et al., 2009; Kessler et al., 2013). Patients often show excessive concern for body size and weight and are prone to emotional instability, impulsivity, and even depression (Heatherton and Baumeister, 1991; Araujo et al., 2010). BED is a serious global public health problem which causes severe psychological and physical distress to patients and seriously affects their life and social work (Husted and Shapira, 2005).

The aim of treatment is to reduce the frequency of binge eating, help patients improve mood (for patients who suffer from severe depression or/and anxiety) and loss weight (especial for patients who are considered selective obesity). There are kinds of intervention approaches including cognitive behavioral therapy (CBT), pharmacologic treatments, psychotherapy and combinations of them. CBT have been the mainstay of BED treatments (Amianto et al., 2015). As clinical research increases, many drugs (such as antidepressants, anticonvulsants, anti-OCD drugs and anti-obesity drugs) have been explored to treat BED (Brownley et al., 2016; McElroy, 2017). In the existing studies, some find Selective Serotonin Reuptake Inhibitor (SSRI) antidepressants have a significant efficacy against BED (McElroy et al., 2000; McElroy et al., 2003), however, others disagree with this finding (Grilo et al., 2005; Stefano et al., 2008). In conclusion, the efficacy of SSRI antidepressants for BED remains controversial, and the purpose of this study is to provide recommendations by assessing the efficacy and acceptability of SSRI antidepressants for patients with BED through a network meta-analysis.

## Methods

### General guidelines of the study

This study was in accordance with the Preferred Reporting Items for Systematic Reviews and Meta-Analyses (PRISMA) guidelines (Supplementary Table 1) (Moher et al., 2015; Shamseer et al., 2015).

### Eligibility criteria

Inclusion criteria were as follows: 1) patients with BED were diagnosed clearly according to the relevant diagnostic criteria of DSM-IV (Segal)/DSM-V (Sarmiento and Lau, 2020), regardless of gender and age; 2) studies were clinically randomized double-blind controlled trials of mono-therapy; 3) patients in SSRI antidepressant groups would be given any kind of SSRI antidepressants, at the same time, patients in the other groups got placebo or other SSRI antidepressants; 4) the results of studies need to include at least one of the following efficacy or acceptability outcomes: the weekly frequency of binge-eating episodes, acceptability (the dropout rate measured by the proportion of patients who dropped out before the study was finished for all causes), Hamilton Rating Scale for Depression (HAMD) score and body weight (kg).

Exclusion criteria were as follows: 1) duplicate publications; 2) conference papers, letters, and reviews; 3) non-monotherapy studies, such as medication combined with psychotherapy or surgery; 4) studies without relevant outcome indicators; 5) studies with incomplete or incorrect data information.

### Search strategy

Two reviewers searched four databases including PubMed, Embase, the Cochrane Library and Web of Science from the earliest record of the databases to July 2022. The detailed search strategy was as follows: (“Binge Eating Disorder” or “Binge-Eating Disorder”) and (“Antidepressants” or “Antidepressant Drugs” or “Antidepressive Agents” or “Antidepressant Medication” or “Fluvoxamine” or “Sertraline” or “Fluoxetine” or “Escitalopram” or “Citalopram” or “Selective Serotonin Reuptake Inhibitor”). The detailed search strategies were presented in Supplementary Table 2. In addition, in order to search for other eligible studies as completely as possible, we searched manually the reference lists of relevant published systematic reviews and meta-analyses.

### Data abstraction

Quality assessment of chosen studies and extraction of data would be accomplished independently by two authors. If there was a disagreement, it would be solved by the third author. In order to facilitate our analysis, we extracted the following items from included studies: the first author’s name, country, publication year of study, mean age of patients, sex ratio, study design, intervention details, treatment duration, drug dose and outcome datas. We extracted outcome data at baseline and post-treatment, as reported in the paper.

### Assessment of bias

Two authors used independently Review manager 5.3 based on the Cochrane Collaboration’s Risk of Bias Tool (Higgins et al., 2011) to evaluate the risk of bias in selected trials. There were seven aspects of bias assessment: 1) random sequence generation (selection bias); 2) allocation concealment (selection bias); 3) blinding of patients and personnel (performance bias); 4) blinding of outcome assessment (detection bias); 5) incomplete outcome data (attrition bias); 6) selective reporting (report bias) and 7) other bias. Outcomes were represented by three colors: green for low risk, yellow for unclear risk, and red for high risk of bias. Disagreements would be decided by the third author.

### Outcomes

The primary outcomes were the changes in binge frequency (the weekly frequency of binge-eating episodes) and acceptability (the dropout rate measured by the proportion of patients who dropped out before the study was finished for all causes). If a study had different follow-up periods, we decided to choose the longest one, because we wanted to pay more attention to long term consequences of antidepressants. The dropout rate was used as a measure for the acceptability of treatments.

The secondary outcomes were the mean changes in HAMD score (HAMD score) and weight (kg) from baseline to endpoint.

### Statistical analysis

The Stata version 16.0 was used to perform this NMA. In this study, we used network diagrams to show direct comparisons between different interventions. Subsequently, we used mean difference (MD) to construct network meta-analysis of continuous data and to estimate the associations of binge frequency, HAMD score and weight changes with interventions. And we used odds ratio (OR) to conduct network meta-analysis of binary data and to estimate the associations of acceptability and interventions. MD < 0 indicated it had a better efficacy in the intervention group. In the same way, OR < 1 indicated better acceptability in the intervention group. I^2^ value was used to evaluate heterogeneity and if I^2^ value exceeded 50%, the degree of heterogeneity was considered to be high. When the degree of heterogeneity was high, we had to choose a random-effects model. Besides, the Surface Under the Cumulative Ranking (SUCRA) was used to reflect the probability order of different antidepressants to be the best treatment option and a higher SUCRA score indicated a more effective or acceptable intervention. In addition, the potential inconsistencies of each loop were evaluated separately by the loop-specific approach and the potential inconsistency of entire NMA was assessed by the design-by-treatment model. If *p* value <0.05, it would be considered as inconsistent. We conducted the comparison-adjusted funnel plot, Egger’s test and Begg’s Test to identify possible bias of publication.

## Results

### Search results

**FIGURE 1:**
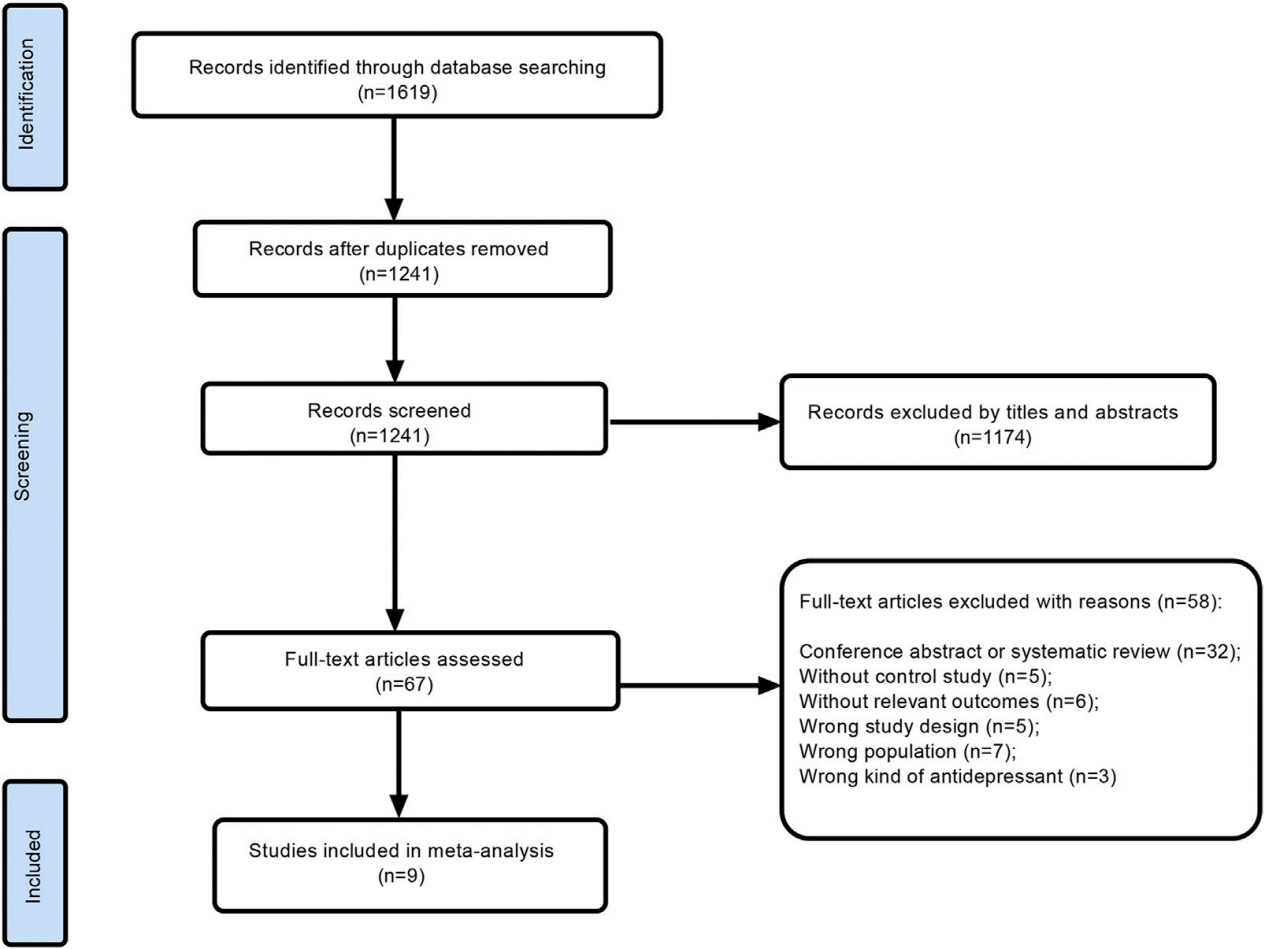
Flow diagram of assessment of studies.

A total of 1619 studies were obtained, including Pubmed (*n* = 131), Embase (*n* = 1126), Cochrane (*n* = 72), and Web of Science (*n* = 290). All the study titles were imported into EndnoteX9 (Bramer, 2018). 378 duplicates were removed. 1241 studies were screened, and we excluded 1174 irrelevant records by titles and abstracts. Subsequently, after searching and thoroughly evaluating 67 articles in the full text, 58 records were excluded. The reasons for the exclusion of 58 papers were as follows: conference abstract or systematic review (*n* = 32), without control study (*n* = 5), without relevant outcomes (*n* = 6), wrong study design (*n* = 5), wrong population (*n* = 7) and wrong kind of antidepressant (*n* = 3). Finally, 9 RCTs (Hudson et al., 1998; McElroy et al., 2000; Ricca et al., 2001; Arnold et al., 2002; McElroy et al., 2003; Pearlstein et al., 2003; Grilo et al., 2005; Guerdjikova et al., 2008; Leombruni et al., 2008) were identified after screening. The detailed process and results of study screening were shown in Figure 1.

### Characteristics of included studies

Ultimately, characteristics of 9 studies were presented in Table 1. The treatment duration of all included trials had a t between 6 and 24 weeks.7 trials were comparisons between SSRI antidepressants and placebo, and 2 trials were direct comparisons between different SSRI antidepressants. Among them, the detailed treatments were as follows: placebo (7 trials), fluvoxamine (3 trials), sertraline (2 trials), fluoxetine (4 trials), escitalopram (1 trial), and citalopram (1 trial). There were 420 participants in this NMA. We could find that the average age of the patients ranged from 25.1 to 44.3. Besides, we noticed that the proportion of women was relatively large in both the treatment group and the control group.

**TABLE 1:** Characteristics of included studies.

Study	Treatment				Control				Duration (weeks)	Outcomes
	Drug	Dose* (mg/d)	Sample size (female/male)	Age*	Drug	Dose* (mg/d)	Sample size (female/male)	Age*		
Hudson et al. (1998)	fluvoxamine	260	42 (39/3)	41.2	placebo	43	43 (38/5)	43	9	b
McElroy et al. (2000)	sertraline	187	18 (16/2)	43.1	placebo	—	16 (16/0)	41	6	ab
Ricca et al. (2001)	fluoxetine	60	21 (12/9)	25.1	fluvoxamine	300	22 (13/9)	26.1	24	b
Arnold et al. (2002)	fluoxetine	71.3	30 (28/2)	41.9	placebo	67.3	30 (28/2)	40.8	6	abcd
Pearlstein et al. (2003)	fluvoxamine	239	9	—	placebo	264	11	—	12	bc
Grilo et al. (2005)	fluoxetine	60	27 (19/8)	44.3	placebo	—	27 (23/4)	43.6	16	b
Guerdjikova et al. (2008)	escitalopram	26.5	21 (21/0)	36.9	placebo	—	23 (22/1)	41	12	abcd
Leombruni et al. (2008)	sertraline	165.9	22 (22/0)	—	fluoxetine	64.5	20 (20/0)	—	24	abd
McElroy et al. (2003)	citalopram	57.9	19 (18/1)	42	placebo	—	19 (18/1)	39.2	6	abcd

HAMD score, Hamilton Rating Scale for Depression score; *, Take the average; —, Not reported in the literature, but all described as comparable; a, binge frequency; b, the all-cause discontinuation rate; c, HAMD score; d, weight.

## Network meta-analysis results

### Primary outcome: Changes in binge frequency

**FIGURE 2:**
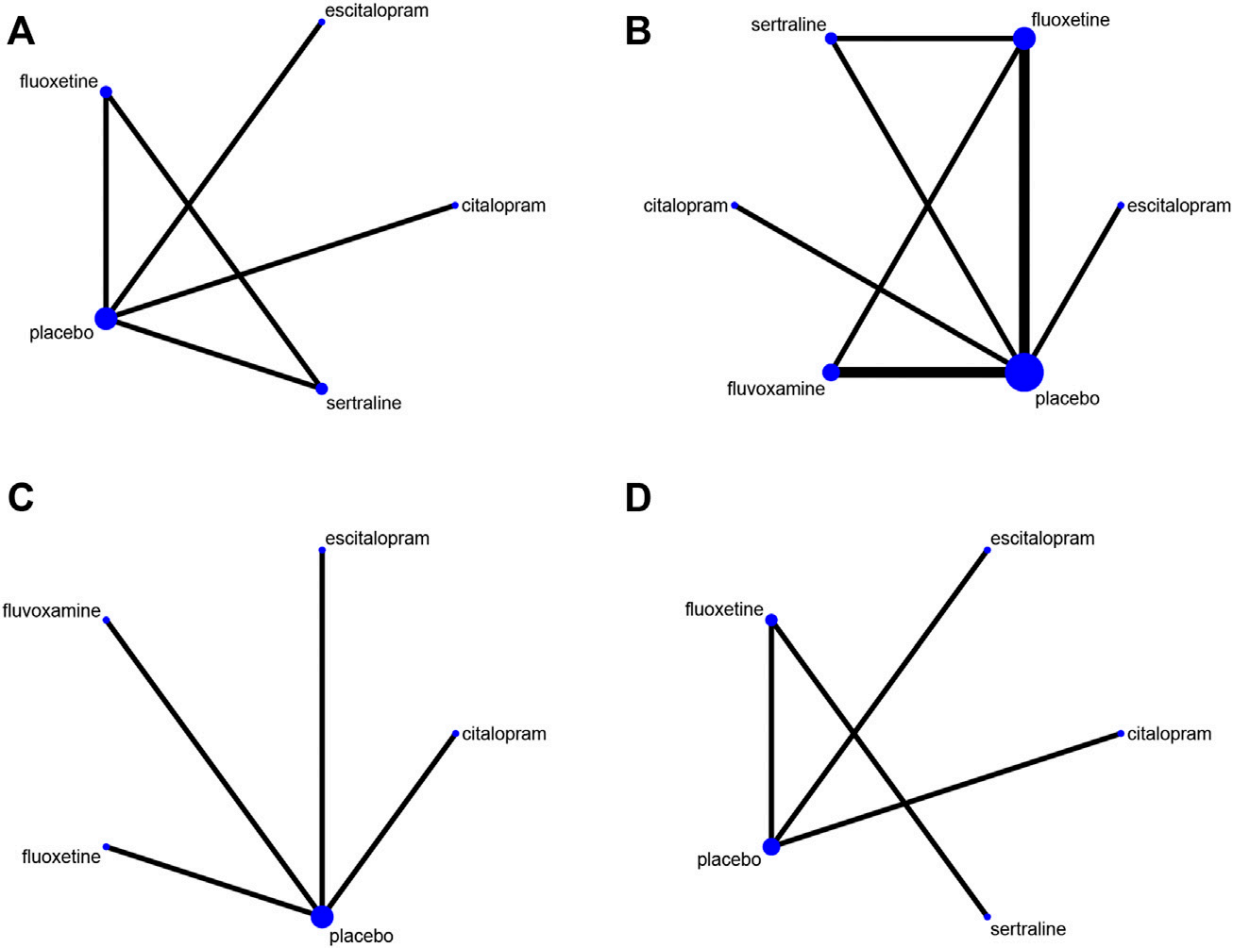
The network plots. **(A)** It was the network plot about the effect of antidepressants on reducing binge frequency of BED patients; **(B)** Dropout rate; **(C)** HAMD score; **(D)** Weight.

**FIGURE 3:**
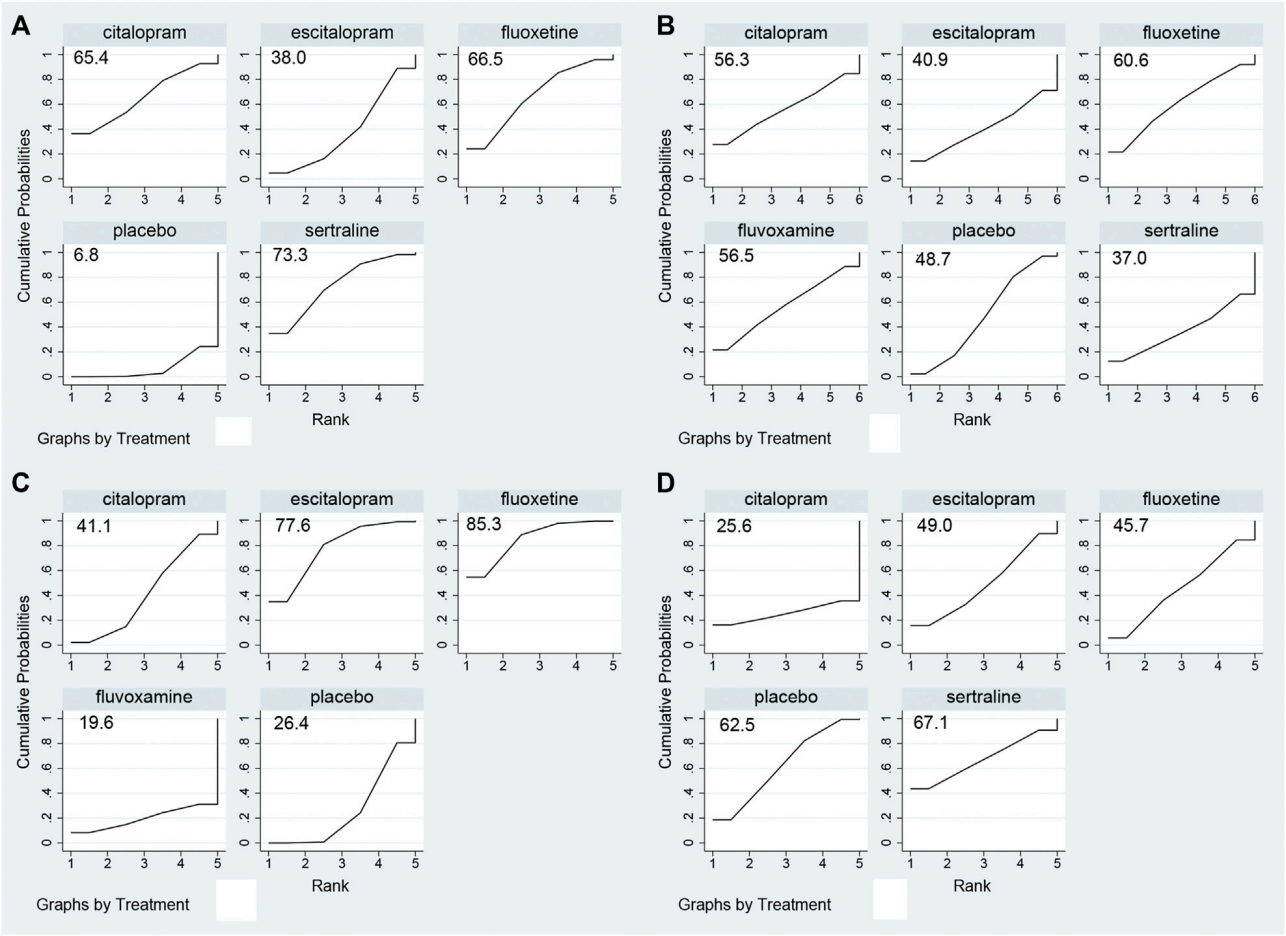
Summary of results from SUCRA. **(A)** Binge frequency; **(B)** Dropout rate; **(C)** HAMD score; **(D)** Weight.

The network plot was shown in Figure 2A. The effect of comparisons among interventions were presented in Table 2. The result of NMA revealed a significant reduction in binge frequency in patients who received sertraline (MD −1.935, 95% CI −3.89 to −0.01) and fluoxetine (MD −1.75, 95% CI −3.77 to −0.02) compared with those who received placebo. Compared to the other three antidepressants, sertraline was proven to reduce binge frequency more effectively. To further understand the results, four antidepressants were ranked by SUCRA values and a higher SUCRA value suggested a lower frequency of binge eating. Figure 3A illustrated that the efficacy ranking in reducing binge frequency was sertraline, fluoxetine, citalopram, escitalopram.

**TABLE 2:** Network Meta-analysis of binge frequency.

Interventions	sertraline	fluoxetine	citalopram	escitalopram	placebo
sertraline	NA	-	-	-	-
fluoxetine	−0.20 (−1.95, 1.55)	-	-	-	-
citalopram	−0.25 (−3.31, 2.80)	−0.05 (−3.16, 3.05)	-	-	-
escitalopram	−1.15 (−3.49, 1.19)	−0.95 (−3.36, 1.45)	−0.90 (−3.60, 1.80)	-	-
placebo	−**1.95 (**−**3.89**, −**0.01)**	−**1.75 (**−**3.77**, −**0.02)**	−1.70 (−4.06, 0.66)	−0.80 (−2.11, 0.51)	NA

The estimate is located at the intersection of the treatments in the column heads and the treatments in the row heads. An MD value <0 indicates that the column-defining treatments got more decrease in binge frequency. An MD value >0 favors the row-defining treatment. Bold results indicate statistical significance. MD, mean difference; NA, not applicable.

### Primary outcome: Dropout rate

The network plot (Figure 2B) included five treatments in the acceptability evaluation. Table 3 showed the ORs and 95%CIs of direct comparisons among different interventions. The NMA found that the dropout rate of included antidepressants was not significantly associated with placebo group. According to SUCRA values (Figure 3B), fluoxetine showed the highest acceptability with a SUCRA value of 60.6. The remaining antidepressants were ranked in the following order: fluvoxamine, citalopram, escitalopram, sertraline.

**TABLE 3:** Network Meta-analysis of dropout rate.

Interventions	Fluoxetine	Citalopram	Fluvoxamine	Escitalopram	Sertraline	Placebo
fluoxetine	NA	-	-	-	-	-
citalopram	−0.15 (−5.02, 4.72)	-	-	-	-	-
fluvoxamine	−0.16 (−4.30, 3.99)	−0.01 (−5.08, 5.07)	-	-	-	-
escitalopram	−0.96 (−5.77, 3.85)	0.27 (−5.38, 5.93)	−0.81 (−6.45, 4.83)	-	-	-
sertraline	−1.24 (−6.12, 3.65)	−1.09 (−6.79, 4.62)	−1.08 (−6.17, 4.01)	1.12 (−5.14, 7.38)	-	-
placebo	−0.50 (−3.25, 2.24)	−0.35 (−4.37, 3.67)	−0.35 (−3.44, 2.75)	−0.02 (−5.73, 5.68)	0.73 (−3.31, 4.78)	NA

The estimate is located at the intersection of the treatments in the column heads and the treatments in the row heads. An OR, value <0 indicates that the column-defining treatments got a lower dropout rate. An OR, value >0 favors the row-defining treatment. Bold results indicate statistical significance. OR, the odds ratio; NA, not applicable.

### Secondary outcome: Changes in Hamilton Rating Scale for Depression score

Figure 2C depicted the network plot for mean changes in the HAMD score. The results (shown in Supplementary Table 3) demonstrated that fluoxetine (MD −2.90, 95% CI −5.45 to −0.35) was linked with a positive drop in HAMD score. The SUCRA value was used to rank the changes in HAMD score among drugs (Figure 3C). In summary, fluoxetine was linked to the greatest reduction in HAMD score, followed by escitalopram, citalopram and fluvoxamine.

### Secondary outcome: Changes in weight

The network was shown in Figure 2D. The NMA revealed that compared to placebo group, all antidepressants were insignificant in losing weight (Supplementary Table 4). The SUCRA value was used to rank mean changes in weight among antidepressants (Figure 3D). In brief, duloxetine was the most helpful for patients wanted to lose weight, other rankings were as follows: sertraline, escitalopram, fluoxetine, citalopram.

### Risk of bias and publication bias

Risk bias of the included studies was presented in Supplementary Figures 1,2. Unclear Risk bias are common due to insufficient method reporting. 3 studies described blinding of outcome assessment while other studies did not describe any details about blinding. Only one of included studies was high risk of bias because this study chose to conceal the detailed generation of random sequence. The most of included trials showed the risk of bias was low. We found it did not exist significant asymmetry in the comparison-adjusted funnel plots and it meant there was no significant bias of publication (Supplementary Figures 3–6), and the results of Egger and Begg Test meant there was no significant bias of publication among the included studies in the NMA (Supplementary Table 5).

### Inconsistency analysis

The NMA did not show any inconsistencies in both global and local inconsistencies, as evaluated by the design-by-treatment methodology and the loop-specific method (Supplementary Table 6).

## Discussion

This study is the first NMA to assess the effectiveness and acceptability of five kinds of SSRI antidepressants in patients with BED. According to the results of this NMA, compared with placebo, both sertraline and fluoxetine were linked with a substantial reduction in binge frequency. Fluoxetine was related with a substantial reduction in HAMD score. Furthermore, in the SUCRA analysis, we found fluoxetine was linked to the second-biggest reduction in binge frequency and the highest reduction in HAMD score. However, all SSRI antidepressants were ineffective in losing weight. As for acceptability, we found there was little difference in dropout rates between antidepressants and placebo, which meant all included antidepressants were generally well-tolerated.

The study’s first major discovery was SSIRs were associated with a significant decrease in both binge frequency and HAMD score in comparison to the placebo control. In some previous studies, serotonin, a neurotransmitter present in the brain, has been linked to the regulation of eating behavior (Blundell, 1986; Leibowitz and Shor-Posner, 1986; Leibowitz, 1988; Curzon, 1990). Serotonergic stimulation which increased the concentration of intrasynaptic serotonin, such as selective serotonin reuptake inhibitors, or directly activate serotonin receptors, such as serotonin receptor agonists, might help consume less food and curb your appetite to eat reduce food consumption (Wurtman and Wurtman, 1979; Wurtman et al., 1985), while administration of receptor antagonists indicated an opposite effect—enhancement of food consumption. Besides, brain serotonin was assumed to have an influence on macronutrient preference *via* satiety systems engaged in eating behavior termination. In addition, it was found in eating disorders, serotonergic dysfunctions might potentially play a role in the emotion (Hudson et al., 1983) and neuroendocrine control problems (Hudson et al., 1982; Gwirtsman et al., 1983). The link between brain serotonergic dysfunction and depression was already discovered (Coppen and Wood, 1982). Another possible explanation for why SSIRs could help in binge episodes reduction was that serotonin was shown to affect negative emotions. There was a study provided clear evidence that unpleasant emotions were crucial in the onset and persistence of binge eating in BED (Dingemans et al., 2017). As was proposed by the theoretical models of BED, poor mood seemed to precede the frequency of binge eating. A study reported that binge eating behavior might be an attempt to down-regulate the emotional distress and reduce dysphoria (Kaye et al., 1988). Therefore, the impact of SSIRs on BED patients could be multidimensional.

In the results, we also found that compared to placebo group, the data of all antidepressants were insignificant in losing weight. The result was same as another meta-analysis written by Vocks S (Vocks et al., 2010). The possible mechanism to explain this result was the reduced basal metabolic rate (BMR) in patients with BED. As some studies reported, bulimia patients had lower calorie requirements for keeping weight, possibly because they had lower-than-normal levels of metabolism-related hormones such as basal thyrotropin level and plasma triiodothyronine level (Devlin et al., 1990; Obarzanek et al., 1991). Furthermore, in the non-resting state, the elevated plasma norepinephrine levels were significantly reduced in bulimia patients compared with healthy individuals (Obarzanek et al., 1991). We suggested that reduced energy needs might be a long-term outcome of abnormal eating practices such fasting, fad dieting, and binge eating, and might be a component in the binge eating syndrome’s continuation. Reduced metabolic rate and a proclivity for quick weight gain might have preceded the development of aberrant eating practices, and future RCTs targeting this topic were needed.

Of course, antidepressants were associated with side effects such as anorexia, gastrointestinal distress, insomnia and nausea. These could affect to some extent the frequency of binge eating and the acceptability of antidepressants in patients with BED. However, there was not detailed reasons for participant withdrawal, such as toxic effects, side effects, participants’ personal reasons, and unexcused loss to follow-up, because we found only a few trials supplied related information. As a result, the drop rate might not just about antidepressants.

The following limits applied to this NMA. First of all, there was a risk of bias if the therapy time was not limited. It was possible that antidepressants might be more effective in the short term than in the long term. However, due to a lack of data, we were unable to find out the correlation between the therapy time and effectiveness. Second, there were some unclear and a high risk of bias in selected trials. It might influence partly results of this study, due to the exhibited methodological shortages. Thirdly, because of the small number of patients and RCTs, the major findings of this NMA should be used with caution in clinical practice.

In conclusion, it is necessary to understand the importance of proper balance between efficacy and acceptability, when choosing a drug. This study gave reliable advice for choosing included five SSRIs to treat BED. As far as both efficacy and acceptability were concerned, fluoxetine might be the best choice. It provided clinicians with medication references.

## Data Availability

The original contributions presented in the study are included in the article/Supplementary Materials, further inquiries can be directed to the corresponding author.
